# Identification and Validation of HOTAIRM1 as a Novel Biomarker for Oral Squamous Cell Carcinoma

**DOI:** 10.3389/fbioe.2021.798584

**Published:** 2022-01-11

**Authors:** Yixiu Yu, Jiamei Niu, Xingwei Zhang, Xue Wang, Hongquan Song, Yingqun Liu, Xiaohui Jiao, Fuyang Chen

**Affiliations:** ^1^ Department of Oral Maxillofacial Surgery, The First Affiliated Hospital of Harbin Medical University, Harbin, China; ^2^ Department of Abdominal Ultrasonography, The First Affiliated Hospital of Harbin Medical University, Harbin, China; ^3^ Pediatric Dentistry Department, The First Affiliated Hospital of Harbin Medical University, Harbin, China; ^4^ Department of Stomatology, The Second Affiliated Hospital of Harbin Medical University, Harbin, China

**Keywords:** oral squamous cell carcinoma, lncRNA HOTAIRM1, biomarker, cell proliferation, cell cycle

## Abstract

ORAL squamous cell carcinoma (OSCC) is a malignant tumor with the highest incidence among tumors involving the oral cavity maxillofacial region, and is notorious for its high recurrence and metastasis potential. Long non-coding RNAs (lncRNAs), which regulate the genesis and evolution of cancers, are potential prognostic biomarkers. This study identified HOTAIRM1 as a novel significantly upregulated lncRNA in OSCC, which is strongly associated with unfavorable prognosis of OSCC. Systematic bioinformatics analyses demonstrated that HOTAIRM1 was closely related to tumor stage, overall survival, genome instability, the tumor cell stemness, the tumor microenvironment, and immunocyte infiltration. Using biological function prediction methods, including Weighted gene co-expression network analysis (WGCNA), Gene set enrichment analysis (GSEA), and Gene set variation analysis (GSVA), HOTAIRM1 plays a pivotal role in OSCC cell proliferation, and is mainly involved in the regulation of the cell cycle. *In vitro*, cell loss-functional experiments confirmed that HOTAIRM1 knockdown significantly inhibited the proliferation of OSCC cells, and arrested the cell cycle in G1 phase. At the molecular level, PCNA and CyclinD1 were obviously reduced after HOTAIRM1 knockdown. The expression of p53 and p21 was upregulated while CDK4 and CDK6 expression was decreased by HOTAIRM1 knockdown. *In vivo*, knocking down HOTAIRM1 significantly inhibited tumor growth, including the tumor size, weight, volume, angiogenesis, and hardness, monitored by ultrasonic imaging and magnetic resonance imaging In summary, our study reports that HOTAIRM1 is closely associated with tumorigenesis of OSCC and promotes cell proliferation by regulating cell cycle. HOTAIRM1 could be a potential prognostic biomarker and a therapeutic target for OSCC.

## Introduction

Oral squamous cell carcinoma (OSCC) is recognized for its high recurrence and metastasis rate, and is the most frequent malignancy in oral tumors, and in particular, presents constantly increasing morbidity (Ferlay et al., 2015; Economopoulou et al., 2017). Although surgeons are constantly adjusting surgical approaches and improving multidisciplinary therapy, the 5-years survival rate of 50% still represents a very poor prognosis, which is related to the specific tissue involved, clinical features, histopathological grading, or other factors (Bray et al., 2018). From another standpoint, the cure rate increases to above 90% through early interventional treatment, in individuals who are diagnosed with early-stage cancer (Lingen et al., 2008; Qiu et al., 2021a). Therefore, it is an urgent task to explore valuable biomarkers, which can provide predictive evidence for the identification of OSCC high-risk groups, early diagnosis, selection of treatment methods, and monitoring of prognosis monitoring.

With the achievements of genome engineering, scientists have no longer been limited to the development of a coding genome, alternatively, they now focus on the non-coding genome, which accounts for 98% of the whole genome (Rong et al., 2021). Long non-coding RNAs (lncRNAs) have emerged as non-coding RNAs, which are defined as a type of RNAs with transcription length not exceed 200 bp, and structural diversity with no coding ability (Salmena et al., 2011). LncRNAs that regulate the expression pattern of mammalian coding genes at the transcriptional or post-transcriptional levels directly or indirectly have been demonstrated on the basis of numerous studies (Winkle et al., 2021). LncRNAs participate in complicated biological processes and are vital regulators in regulating or deregulating expression of genes that determine the cellular fate. LncRNAs have been identified to be involved in cancer formation and development in thyroid cancer, head and neck cancer, pancreatic cancer, and glioma (Ahadi 2020; Song and Kim 2021). Studies have confirmed that abnormal expression of some functional lncRNAs could affect the progression of cancer. For example, LINC00941 is abnormally upregulated in pancreatic cancer, promoting glycolysis by regulating the Hippo pathway, thereby promoting the malignant biological behavior of pancreatic cancer (Xu et al., 2021a). H19 is a significantly down-regulated lncRNA in prostate cancer confirmed to inhibit the invasion of tumor cells by targeting TGFBI via regulating miR-675, which could be a biomarker to benefit diagnosis and therapy of advanced prostate cancer (Zhu et al., 2014). Thus, it is of the utmost importance to explore the expression, distribution, and molecular biological function of OSCC-related lncRNAs as valuable markers in diagnosis, treatment, and prognosis. It has been reported that the expression of HOTAIRM1 is evidently downregulated in lung adenocarcinoma tissues, and the growth of H1650 and PC-9 cell lines were accelerated by promoting cell-cycle progression when HOTAIRM1 was silenced (Chen et al., 2020). In glioblastoma (BGM), researcher confirmed the upregulation of HOTAIRM1, and further demonstrated that HOTAIRM1 acted as a facilitator of malignant-behavior in BGM, which was shown to accelerate the occurrence of migration and invasion (Xie et al., 2020). It’s a sign that HOTAIRM1 may be a cancer-associated lncRNA and influences the malignant progression of some cancers. Nevertheless, the expression pattern and molecular function of HOTAIRM1 in OSCC have not been elucidated to date.

In our study, HOTAIRM1 was identified as an upregulated lncRNA and was found to be closely associated with OSCC overall survival (OS), tumor stage, genomic instability, the tumor cell stemness, the tumor microenvironment (TME), and immune inflammation. In addition, our *in vitro* studies revealed that HOTAIRM1 could accelerate the cell proliferation by regulating the cell cycle of OSCC cells. *In vivo*, the tumor growth was significantly inhibited by HOTAIRM1 knockdown, including tumor size, weight, volume, angiogenesis, and tumor hardness, as assessed by ultrasonic imaging (USI) and magnetic resonance imaging (MRI). In conclusion, our findings have demonstrated that HOTAIRM1 is a risk factor for OSCC, and confirmed its function, and we suggest that HOTAIRM1 could be a novel biomarker for the early diagnosis and therapy of OSCC.

## Materials and Methods

### Data Extraction

The data used for our analysis was obtained from TCGA-HNSC dataset, which can be downloaded through the Genomic Data Commons (GDC) (https://portal.gdc.cancer.gov/), and including the following information: Exp (expression data gathered in HTSeq-Counts, HTSeq-FPKM, copy number variation (CNV) and somatic mutations data. From the UCSC Xena database (https://xenabrowser.net/), we obtained the clinical phenotype and survival data. Patient samples with lesion sites in the oral cavity (lip, gum, palate, jaw, floor of mouth, tongue, maxilla, mandible, and buccal mucosa) were selected for our study and those without clinical data information were excluded.

### Data Processing and Differential Expression Analysis

Firstly, the genes whose 80% of samples’ count value was at least one were retained for the following analyses. Meanwhile, the human gene transfer format (GTF) annotation file was acquired from the GENCODE project (http://www.gencodegenes.org, release 35) to convert the Ensembl gene ID into the gene symbol and extracted the lncRNAs and protein-coding genes profiles. Then, 4324 lncRNAs and 15952 mRNAs and their corresponding count and FPKM expression profiles were obtained.

Secondly, differential expression analysis of lncRNAs between OSCC and control samples was performed using the R “DESeq2” package. It was considered to be significant when DEGs meeting the following conditions: I. false discovery rate (FDR) < 0.01; II. |log2Fold Change (FC)|> 1 (Zhao and Ruan 2020; Xu et al., 2021b; Markert, et al., 2021). Here, we focused on the lncRNA HOTAIRM1, who is associated with multiple cancers, for subsequent analysis. The samples were divided into high- and low-HOTAIRM1 groups after the median HOTAIRM1 expression value (1.5948) was set as the cut-off point (Ozawa et al., 2017).

### Survival Analysis

The R package “survival” and “survminer” were used to perform univariate and multivariate Cox regression analyses and Kaplan-Meier survival analysis. To retian the samples with complete clinical data, we screened 288 samples from 324 OSCC patients for overall survival (OS) analysis. The low and high groups were cut-off through the median of HOTAIRM1 expression value. *p*-value < 0.05 was considered statistically significant based on log-rank test.

### Genomic Variation Analysis

Firstly, the CNV data relevant to HOTAIRM1 expression were analyzed using “gistic2.0” software, setting 0.1 as the *q*-value, and was specific to chromosomes alterationsbetween HOTAIRM1-high and HOTAIRM1-low groups; the results are presented using the R package “maftools”. Secondly, to compare the tumor mutation burden (TMB) between HOTAIRM1-high and HOTAIRM1-low groups, the somatic raw variant counts identified by TCGA were calculated, while whole-exome 38 Mb size was regarded as the estimate. Finally, to analyze the correlation between the HOTAIRM1 and genetic mutation, the Pearson correlation coefficient (PCC) was calculated on the HOTAIRM1 expression and genetic mutations vector. It is considered to be significantly correlated when *p*-value of PCC< 0.05.

### Correlation Analyses Between Tumor Microenviroment and HOTAIRM1

For analyzing the association between tumor microenvironment (TME) and HOTAIRM1 expression, the R packages “ESTIMATE” was employed to get the stromal score, immune score and ESTIMATE score (Yoshihara et al., 2013). And, to assess the state of infiltration of immune cells, the CIBERSORT algorithm was used to analyze (Newman et al., 2015). The PCC was conducted to analyze the correlation between HOTAIRM1 and tumor cell stemness, TME and immune-infiltrating cells. A significant correlation was considered to be when *p*-value of PCC< 0.05.

### Weighted Gene Co-Expression Network Analysis

According to absolute median difference of expression values, the first 5000 mRNAs in all the OSCC samples were selected to conduct WGCNA using the R package “WGCNA” (Langfelder and Horvath 2008) to aquire the gene-sets associated with HOTAIRM1. The power parameters were selected through the “pickSoftThreshold.” We use topology overlap similarity (TOM) matrix to represent the similarity of two genes in network structure and modules containing at least 30 genes were retained. We next calculated the correlation coefficient between the modules and HOTAIRM1 expression to determine the modules most related to HOTAIRM1. To identify the key genes in this module having gene significances (GS) ≥0.3, the module membership (MM) in the top 10% after calculating were obtained.

Next, we further analyzed the biological functions involved in these key genes. Gene Ontology (GO) and Kyoto Encyclopedia of Genes and Genomes (KEGG) analysis were completed by the R package “ClusterProfiler.” After the Benjaminiand Hochberg (BH) correction, *p*-values< 0.05 were considered to indicate significantly enriched function and the results were displayed by R package “ggplot2” and “enrichplot”, respectively. In addition, we obtained the protein-protein interaction (PPI) information of key genes from the STRING database (https://string-db.org/) and visualized the PPI network using “cytoscape” software. In this study, the interaction pairs with combined score> 0.4 were retained.

### Analysis of Biological Function Associated With HOTAIRM1 Up-Regulation

To further identify the biological changes caused by abnormal HOTAIRM1 expression, we choose the top 20% samples of the high-HOTAIRM1 group (highest 20% samples) and the bottom 20% samples of the low-HOTAIRM1 group (lowest 20% samples) to analyze. The rank of each mRNA was determined based on their Fold Change value (DESeq2) in the above samples. Meanwhile, the gene sets of Hallmark, KEGG pathways, GO-BP terms and Reactome pathways were all collected from Molecular Signatures Database (MSigDB). Then, applying the R software package “clusterProfiler” to conduct Gene Set Enrichment Analysis (GSEA), and the gene sets were significant with adjust *p*-values (BH) < 0.05. Finally, to confirm that HOTAIRM1 was indeed involved in the biological processes of the cell cycle and cell proliferation in OSCC, the Gene Set Variation Analysis (GSVA) was conducted in low- and high- HOTAIRM1 expression groups. This process was completed by R “GSVA” package.

### Cell Culture

Human OSCC cell lines (Cal27 and SCC9) were obtained from the Harbin Medical University (Harbin, China), and human oral epithelial cells (HOEC) were originally supplied by the American Type Culture Collection (ATCC, United States). Cal27 and HOEC cells were maintained in a 37°C incubator with humidified atmosphere containing 5% CO_2_ and cultured with Dulbecco’s Modified Eagle’s Medium (DMEM) containing 10% Fetal Bovine Serum (FBS), while Roswell Park Memorial Institute-1640 (RPMI 1640) containing 10% FBS was used for SCC9 cells.

### Cell Transfection

Specific sh-RNAs (sh-HOTAIRM1) and the control sh-RNAs (sh-NC) were obtained from General Biosystems (Anhui, China). The sh-RNAs were transfected using the Lipofectamine 3000 reagent (following the manufacturer’s instructions) to knockdown HOTAIRM1 expression in Cal27 and SCC9 cells.

### Quantitative Real-Time Polymerase Chain Reaction

Total RNA of OSCC cells was isolated using a Total RNA Extraction Kit (Suzhou, China) and then transcribed into cDNA using Prime Script RT Reagent Kit (Takara, Shiga, Japan). Subsequently, the SYBR Green Master Mix (TOYOBO, Japan) was applied to conduct quantitative real-time polymerase chain reaction (qRT-PCR) on the ABIPRISM 7900HT instrument (Applied Biosystems, United States). The above experiments were repeated 3 times. Primer sequences used were as follows:

HOTAIRM1
  F: 5′-TTG​ACC​TGG​AGA​CTG​GTA​GC-3′
  R: 5′-TTC​AGT​GCA​CAG​GTT​CAA​GC-3′;
β-actin
  F:5′ ATG​AAC​TGG​CGA​GAG​GTC​TGT3′
  R:5′ CCA​GGA​ATG​AGT​AAC​ACG​GAG​T3′.

### Cell Counting Kit-8 Assay

The Cell counting kit-8 (CCK-8) (Dojindo, Rockville, MD, United States) was used to estimate the proliferation ability. The CCK-8 solution (10 μL) was added at specified culture time points (24, 48, 72, and 96 h), to each well of Cal27 and SCC9 cells seeded in a 96-well plate (density: 2.0 × 10^3^ cells/well), and then cultured for an additional 2 h at 37°C in the dark. Finally, the optical absorption value, which represented the number of viable cells, was evaluated at 450 nm.

### Colony Formation Assay

One thousand treated OSCC cells were plated in a culture dish whose diameter was 6-cm. After a 2-week culture, visible colonies were fixed with methanol after staining with 0.5% crystal violet for 15 min, and were counted using a microscope (IX51, Olympus, Tokyo, Japan).

### Western Blotting

First, the total proteins were extracted from the cells using the radioimmunoprecipitation assay buffer (Pierce, Rockford, IL, United States). The proteins at equivalent amount were employed for the sodium dodecyl sulfate-polyacrylamide gel electro-phoresis (SDS-PAGE), and then transferred to the polyvinylidene fluoride membranes. After blocking membranes with 5% non-fat milk/TBST for 1 hour, incubation with the primary antibodies, including PCNA, Cyclin D1, CDK4, CDK6, *β*-actin (Proteintech, Wuhan, Hubei, China), p53 and p21 (Cell Signaling Technology, Danvers, MA, United States), were performed at 4°C overnight. Next, blots were exposed to the corresponding horseradish per-oxidase-conjugated secondary antibodies for an additional hour at room temperature. The secondary antibodies used in this study, including anti-mouse IgG and anti-rabbit IgG, were provided by Proteintech (Wuhan, Hubei, China). Finally, images of protein bands were obtained using the BioSpectrum 600 Imaging System (UVP, United States).

### Cell Cycle Analysis

Transfected OSCC cells were washed twice by cold PBS and fixed using precooled 75% ethanol. Thereafter, the cell-cycle phase distribution of samples that had been digested using RNase (10 mg/ml) and stained using propidium iodide (PI, 1 mg/ml), were detected, and analyzed on FACSCalibur flow cytometer (BD Biosciences, United States). The above process was repeated 3 times.

### Animal Experiments *in vivo*

The female BALB/c nude mice (aged, 4–5 weeks) were purchased from Charles River Japan (Beijing, China) and maintained in a sterile environment. This animal experiment was approved by the Committee on Animals of the First Affiliated Hospital of Harbin Medical University (Harbin, China). After animals were anesthetized, Cal27 cells (sh-NC or sh-HOTAIRM1) were injected subcutaneously into the bilateral flanks of BALB/c nude mice. For each mouse, the OSCC cells transfected with sh-NC were inoculated into the left flank, while the OSCC cells transfected with sh-HOTAIRM1 were inoculated into the right flank. Every week, the animal models were weighed and measured, and subjected to USI and MRI to observed neoplastic formation and growth, for a total treatment period of 4 weeks. The tumor volume was calculated using the following formula: V = 0.5 × length × Width^2^. At the end of the experiment, the tumors in mice were excised after the animals were euthanized.

### Ultrasonic Imaging

The Ultrasound System (Aplio 500, Canon, Japan) was applied to observe the tumors *in vivo* by USI. The specific imaging modes used were B-mode ultrasonography to evaluate neoplastic growth, Color doppler flow imaging (CDFI) and Color Power Angio (CPA) were used to evaluate angiopoiesis and Ultrasonic elastosonography (USE) to evaluate the tumor stiffness.

### Magnetic Resonance Imaging

MRI soft tissue imaging was performed using an Achieva 3.0T TX MRI System (Philip, Netherlands) to observe the neoplastic growth and signal intensity with clinical oral floor imaging sequences, containing T1WI, T2WI and T2-FLAIR images.

### Statistical Analysis

SPSS software (Version 22.0, Armonk, NY, United States) was employed to conduct the statistic analysis. All the experimental data, which were obtained after three repetitions of independent experiments, were presented as means ± standard deviation (SD). To compare the difference between two groups, the Student’s *t*-test was performed, and one-way ANOVA was conducted to compare the difference between multiple groups. It was regarded as statistically significant when *p-*values < 0.05.

## Results

HOTAIRM1 was identified as abnormally upregulated in OSCC and was associated with tumor stage and worse prognosis

**FIGURE 1:**
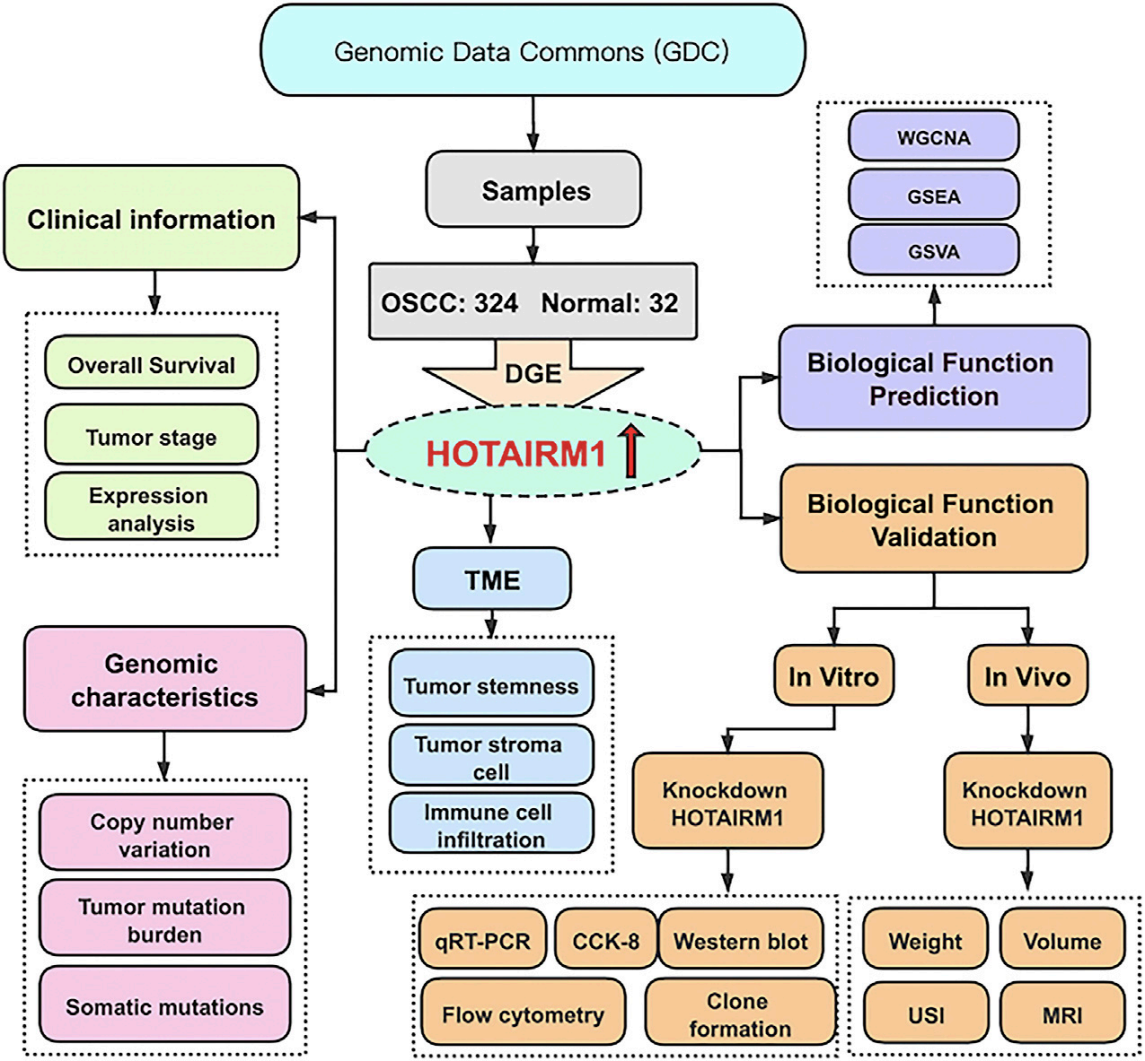
The flowchart of this study.

**FIGURE 2:**
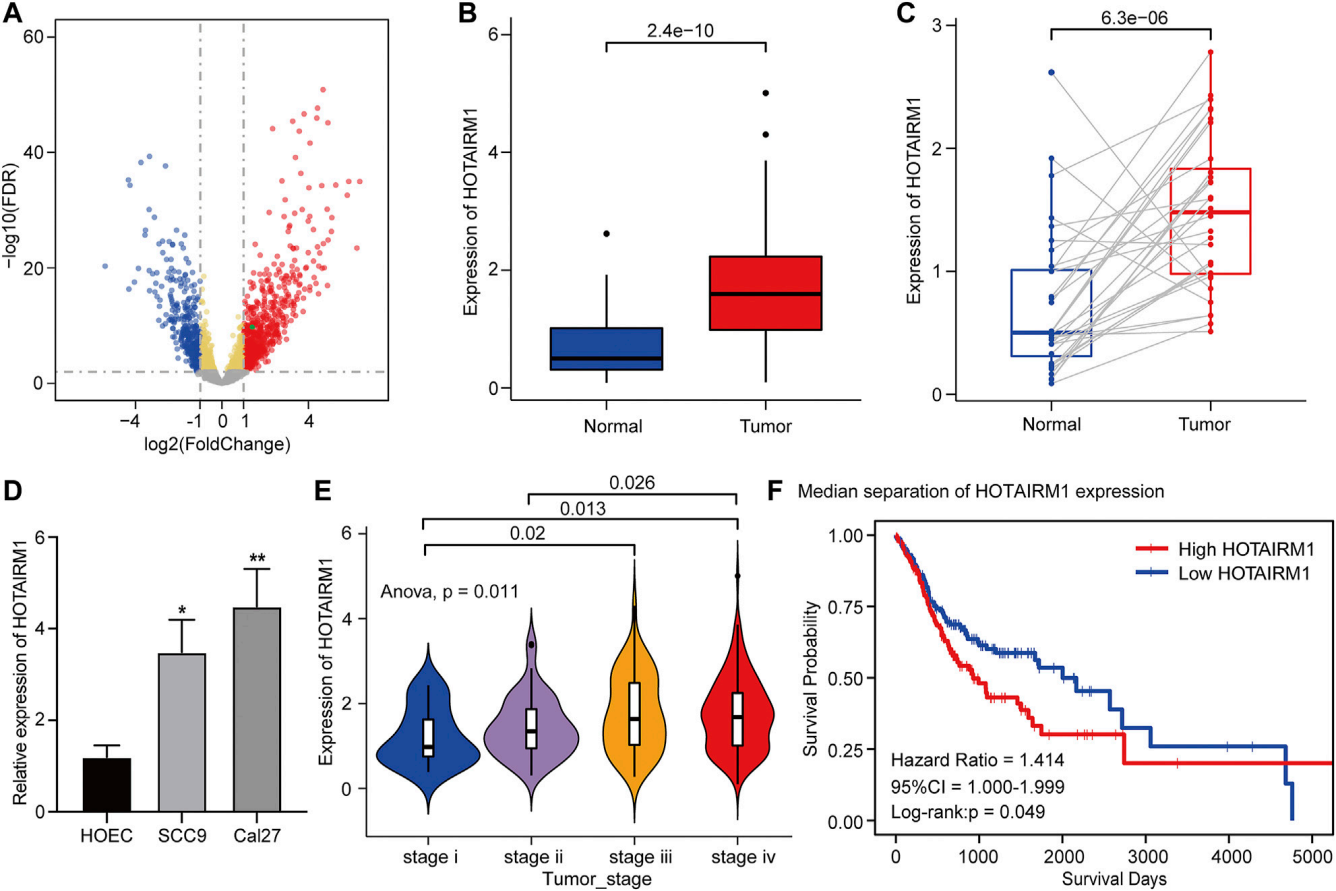
Upregulation and clinical significance of HOTAIRM1 in OSCC. **(A)** Volcano plot for DEGs. The blue represented the significantly down-regulated genes; the red dots represented the significantly up-regulated genes; the green dot was the HOTAIRM1. **(B)** The comparison of HOTAIRM1 expression between all OSCC samples and control samples. **(C)** The comparison of HOTAIRM1 expression between OSCC tissues and corresponding adjacent normal tissues. **(D)** qRT-PCR analysis confirming HOTAIRM1 upregulation in OSCC cell lines. **(E)** Correlation analysis between HOTAIRM1 expression and OSCC tumor stage indicating the higher HOTAIRM1 expression was associated with a higher tumor stage. **(F)** Kaplan-Meier analysis suggested that high HOTAIRM1 expression was associated with unfavorable prognosis in OSCC. *p < 0.05, **p < 0.01.

Figure 1 shows the complete workflow of our study to explore LncRNA HOTAIRM1 in OSCC. According to the conditions in “Data Extraction” screening, we finally acquired 342 OSCC patients and a control group comprised of 32 para-cancerous samples (Supplementary Table S1). Following calculation and processing of the sample data, finally, 4324 lncRNAs and 15,952 mRNAs were obtained. Then the obtained lncRNAs were employed for the DEG analysis. Eventually, we identified 1069 abnormally expressed lncRNAs in the OSCC dataset (Supplementary Table S2), which are illustrated in the volcano plot in Figure 2A, including 717 upregulated lncRNAs and 352 downregulated lncRNAs. Among the DEGs, HOTAIRM1 was observed to be a markedly upregulated lncRNA, which has not been reported in previous studies on OSCC. Further, we found that HOTAIRM1 was indeed highly expressed in cancer samples but not in normal samples, even in the paired-samples comparison (Figures 2B,C). In addition, the expression of HOTAIRM1 in OSCC cell lines (SCC9 and Cal27) was also significantly higher than in human oral epithelial cells (HOEC), as assessed by qRT-PCR (Figure 2D). The power of prediction by HOTAIRM1 as a potential biomarker was evaluated by a receiver operating characteristic (ROC) curve and the area under the ROC (AUC). The result showed that the AUC value was 0.838, indicating the excellent power of prediction by HOTAIRM1 as a potential biomarker (Supplementary Figure S1). Next, we conducted an analysis to evaluate the relationship between OS and clinical data, including HOTAIRM1 expression, age, gender, tumor stage, smoking as variables. In the univariate Cox-regression analysis, HOTAIRM1 expression and tumor stage were revealed as the risk factors for OSCC patient prognosis. While, in the multivariate Cox-regression analysis, age, HOTAIRM1 expression and tumor stage were all significantly independent predictors of OS of OSCC patients (Table 1). Thus, it was reasonable to suspect that the elevation of HOTAIRM1 may be a risk factor for OSCC. In addition, we identified a correlation between HOTAIRM1 expression and tumor stage, indicating that more advanced OSCC stage was associated with the higher expression of HOTAIRM1 (Figure 2E). With regard to OS analysis, OSCC patients with high-HOTAIRM1 expression were characterized by a low survival rate and poorer bleak prognosis (Figure 2F). Combined with the above findings, we considered HOTAIRM1 as a risk factor likely to promote tumorigenesis of OSCC.

**TABLE 1:** Univariate and multivariate Cox regression analyses for overall survival in OSCC patients.

Variables	Status	Number	Univariate analysis			Multivariate analysis		
			DSBA	95% CI of HR	Pvalue	HR	95% CI of HR	P- value
HOTAIRM1	low/high	146/142	1.414	1.000–1.999	0.049^a^	1.458	1.019–2.087	0.039^a^
Age	≤60/>60	126/162	1.314	0.924–1.870	0.129	1.505	1.031–2.197	0.034^a^
Gender	female/male	90/198	0.943	0.655–1.359	0.754	0.940	0.639–1.382	0.753
Tumor stage	stage I	18	1 (ref)	—	—	1 (ref)	—	—
	Stage II	51	1.9773	0.584–6.693	0.273	1.738	0.510–5.921	0.377
	Stage III	60	2.6724	0.801–8.920	0.110	2.231	0.663–7.507	0.195
	Stage IV	159	4.2115	1.329–13.343	0.015^a^	3.637	1.137–11.658	0.030^a^
Smoking	no/yes	197/91	1.3787	0.956–1.989	0.086	1.230	0.839–1.803	0.288

a *p* < 0.05.

### Upregulation of HOTAIRM1 Was Associated With Genomic Instability

**FIGURE 3:**
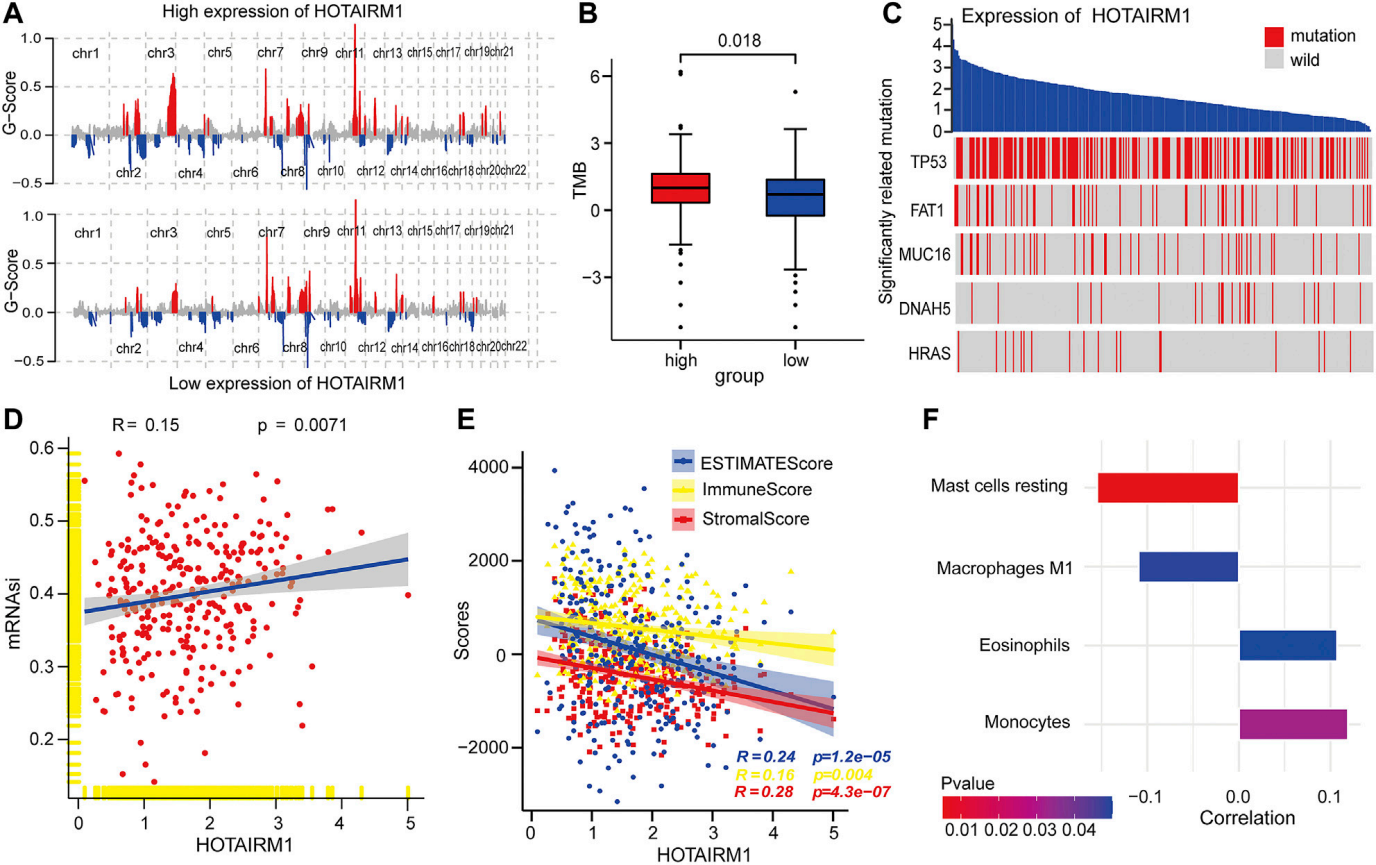
Correlation analyses between HOTAIRM1 and tumorigenesis-relevant variables. **(A)** The copy number variation of each chromosome in the samples with high-HOTAIRM1 and low-HOTAIRM1 expression of OSCC is shown. The red peaks represent recurring focal amplification of chromosomes, while the blue areas represent deletions. **(B)** TMB was higher in OSCC patients with high-HOTAIRM1 expression than in patients with low-HOTAIRM1 expression. **(C)** Relevance between HOTAIRM1 expression and status of gene mutations and the five most correlated genes. **(D)** The relative association between the stemness index and HOTAIRM1 expression in OSCC. **(E)** The correlation analysis between HOTAIRM1 expression and cellular components related to the tumor microenvironment in OSCC, as assessed by the stromal score, immune score, and ESTIMATE score. **(F)** Several immune-infiltrating cells markedly associated with HOTAIRM1 expression.

To explore the inherent factors associated with abnormal HOTAIRM1-expression and OSCC carcinogenesis, we investigated different genomic alterations in terms of somatic variation and CNVs. First, we evaluated the copy number variation status between groups with high-HOTAIRM1 and low-HOTAIRM1 levels (Figure 3A), and determined that amplification of CHR3 and deficiency of CHR2 were significant variations in the high-HOTAIRM1 expression group, whereas in the low-HOTAIRM1 expression group, CHR11 amplification and the CHR13 deficiency were observed. These findings associated HOTAIRM1 expression to CNVs of specific genes. Next, we evaluated tumor mutation burden (TMB) status. In the high-HOTAIRM1 group, the TMB was evidently higher compared to that in the low-HOTAIRM1 expression group (Figure 3B), which indicated that non-synonymous mutations may be involved in the dysregulation of HOTAIRM1 (*t*-test, *p* < 0.05). To better understand the underlying link between genetic mutations and the dysregulation of HOTAIRM1, we identified the first five most relevant mutated genes (TP53, FAT1, MUC16, DNAH5, HRAS), which provided evidence for the association between genome aberrations and upregulation of HOTAIRM1 in OSCC (Figure 3C).

### HOTAIRM1 Expression Was Related With the Tumor Cell Stemness and Tumor Microenvironment

To explore the relationship between HOTAIRM1 expression and tumor cell dryness, pearson correlation analysis was performed between HOTAIRM1 expression and stemness indices from mRNA level (mRNAsi). The results showed that the higher HOTAIRM1 expression was, the higher mRNAsi was (Figure 3D), implying that HOTAIRM1 may contribute to the progression of OSCC (*p* = 0.0071). It is well known that tumors and the TME are an indivisible whole, therefore, we analysed the association between HOTAIRM1 and stromal cells, and immunocyte infiltration. The pearson correlation analysis showed that HOTAIRM1 expression level was negatively correlated with Stromal Score, ESTIMATE Score and Immune Score (Figure 3E). With regard to immune-infiltrating cells, there were four closely related immune cells associated with HOTAIRM1 expression. Among these, there was a negative correlation between HOTAIRM1 expression and resting mast cell and M1 macrophages levels, while a positive correlation was observed between HOTAIRM1 expression and eosinophils and monocytes infiltration (Figure 3F). The above results suggested that HOTAIRM1 might facilitate tumor cell proliferation in OSCC.

### Biological Functions Related to HOTAIRM1 in ORAL Squamous Cell Carcinoma

**FIGURE 4:**
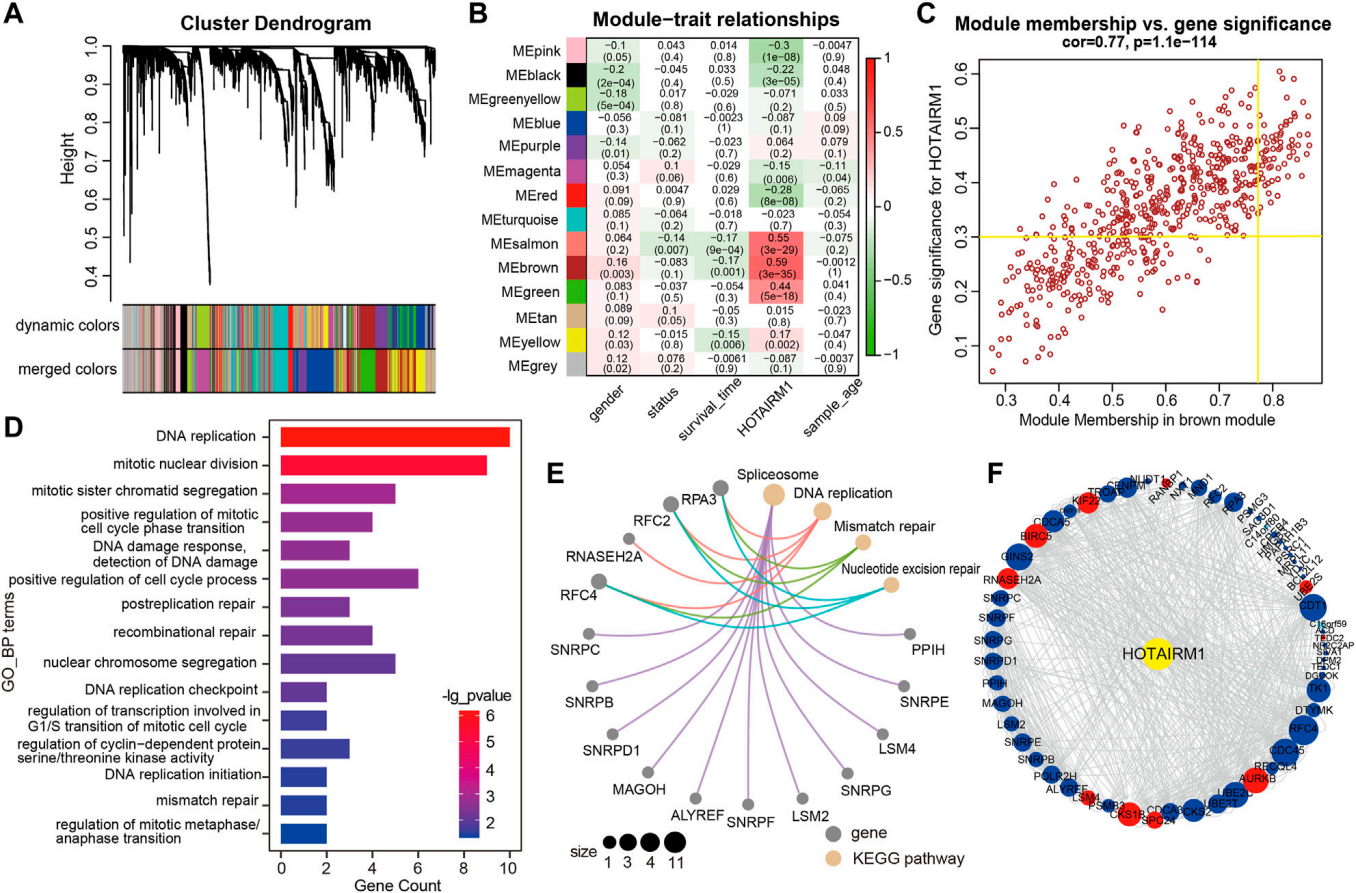
WGCNA module analysis showing that HOTAIRM1 was mainly involved in the biological process of cell proliferation. **(A)** A total of 14 related modules were identified through WGCNA, represented by different colors, which were displayed in a hierarchical clustering tree diagram. **(B)** The heatmap showed the correlation between HOTAIRM1 expression and the modular eigengenes. The brown module showed the most positive significant correlation with HOTAIRM1. **(C)** Scatter plot of module eigengenes in the brown module. The genes meeting the module criteria, *p*-value ≥ 0.3 and in the top 10% of the brown module, are identified as key driving genes associated with high HOTAIRM1 expression. **(D)** Functional enrichment analysis by GO demonstrated that key driver genes related to HOTAIRM1 were enriched in cell proliferation processe. **(E)** The KEGG signaling pathway analysis showing driver genes related to HOTAIRM1 expression enriched in the pathways regulating cell proliferation. **(F)** The interrelationship between the driver genes illustrated in the PPI network.

WGCNA was used to screen genes interacting with HOTAIRM1 in OSCC. A total of 14 modules were obtained following data processing (Figure 4A), in which the brown module was identified as the most significantly related to HOTAIRM1 as it presented the highest Pearson’s coefficient, which suggested signifying that HOTAIRM1 may could regulate or be regulated by these genes in brown module (Figure 4B). A total of 57 key genes whose expression was recognized as being influenced by HOTAIRM1 and were used to speculate the functions of HOTAIRM1 in OSCC (Figure 4C). Following functional enrichment analysis on these key genes, the 57 genes were determined to be active in biological processes regulating cell proliferation, including DNA replication, mitotic nuclear division, and changes in cell cycle phases (Figure 4D). In the KEGG pathways analysis, the DNA replication, Mismatch repair, and Nucleotide excision repair pathways were significantly enriched and were associated with cellular proliferation (Figure 4E). The above results indicated that HOTAIRM1 was involved in OSCC-cell proliferation and specifically in cell cycle regulation. A PPI-network-analysis was performed, which demonstrated the interactive relationship between the 57 critical genes, which supported the functional prediction regarding HOTAIRM1 activity in OSCC (Figure 4F). In addition, we retained the interaction pairs with combined score> 0.4 (Supplementary Table S3).

### Gene Set Enrichment Analysis and Gene Set Variation Analysis Indicated HOTAIRM1 Promoted Cell Proliferation

**FIGURE 5:**
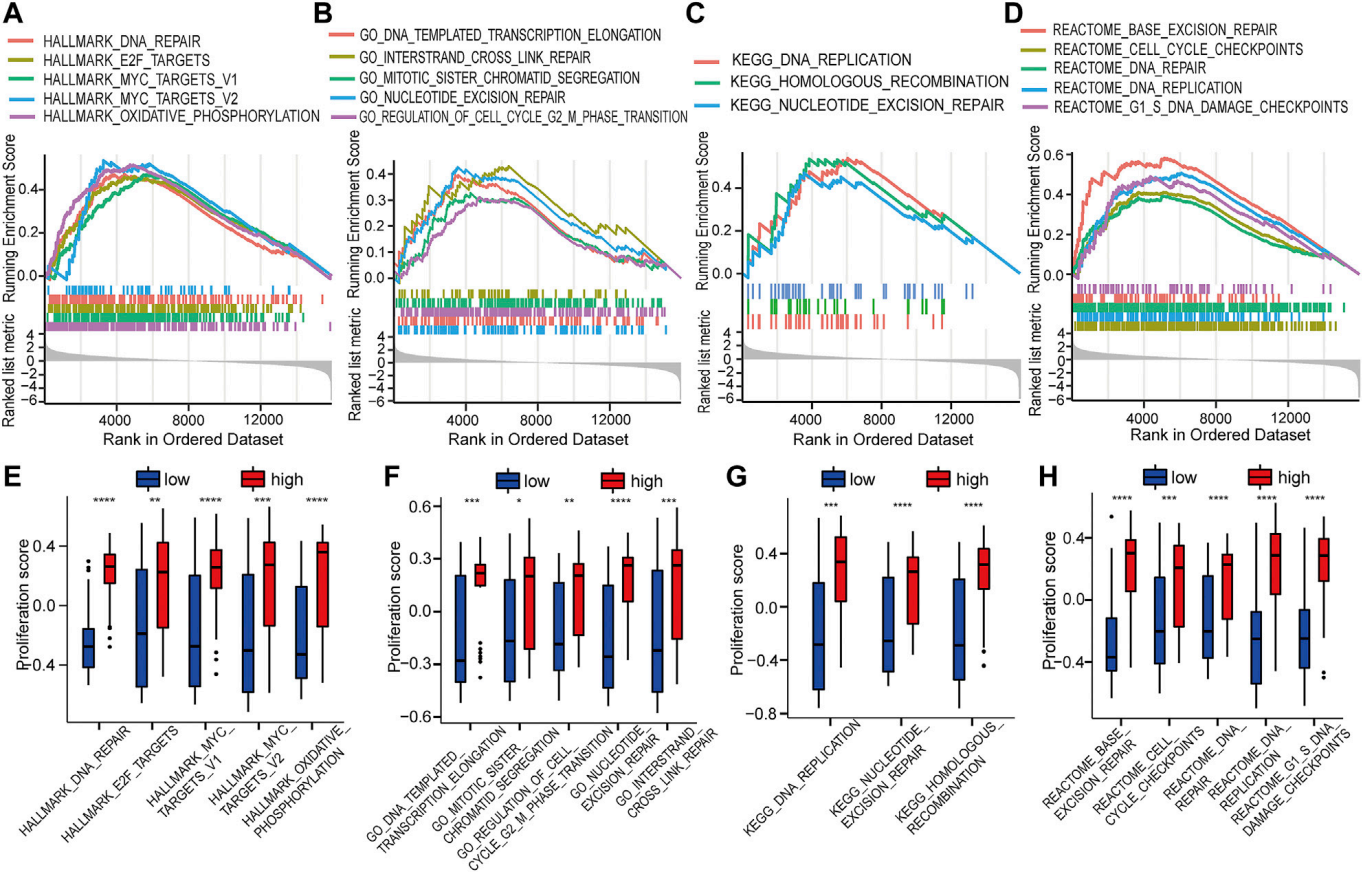
The function of HOTAIRM1 mainly involves cell proliferation in OSCC further indicated by GSEA and GSVA analyses. **(A)** GSEA identified the gene sets significantly enriched in Hallmark. **(B)** GSEA identified the gene sets significantly enriched in GO. **(C)** GSEA identified the gene sets significantly enriched in KEGG. **(D)** GSEA identified the gene sets significantly enriched in REACTOME. **(E–H)** The scores related to cell proliferation assessed in the GSVA in the high-HOTAIRM1 expression group was higher than in the low expression group, suggesting the involvement of HOTAIRM1 in regulating cell proliferation. *p < 0.05, **p < 0.01, ***p < 0.001, ****p < 0.0001.

GSEA was used to identify the potential biomolecular alterations caused by up-regulation of HOTAIRM1, focusing on the gene sets of Hallmark, GO, KEGG, and REACTOME. The gene sets enriched were mostly related to cell proliferative processes, for example, DNA repair and E2F targets in Hallmark (Figure 5A), regulation of the cell cycle G2/M phase transition and nucleotide excision repair in GO (Figure 5B), DNA replication and homologous recombination in KEGG (Figure 5C), and cell cycle checkpoints and G1/S DNA damage checkpoints in REACTOME (Figure 5D). GSVA was conducted to further confirm the GSEA results by comparing the cellular proliferation activity between groups with high-HOTAIRM1 and low-HOTAIRM1 expression, and a more active state of cellular proliferation was observed in high-HOTAIRM1 expression group (Figures 5E–H).

### Knockdown of HOTAIRM1 Inhibited Proliferation and Arrested the Cell Cycle of ORAL Squamous Cell Carcinoma Cells

**FIGURE 6:**
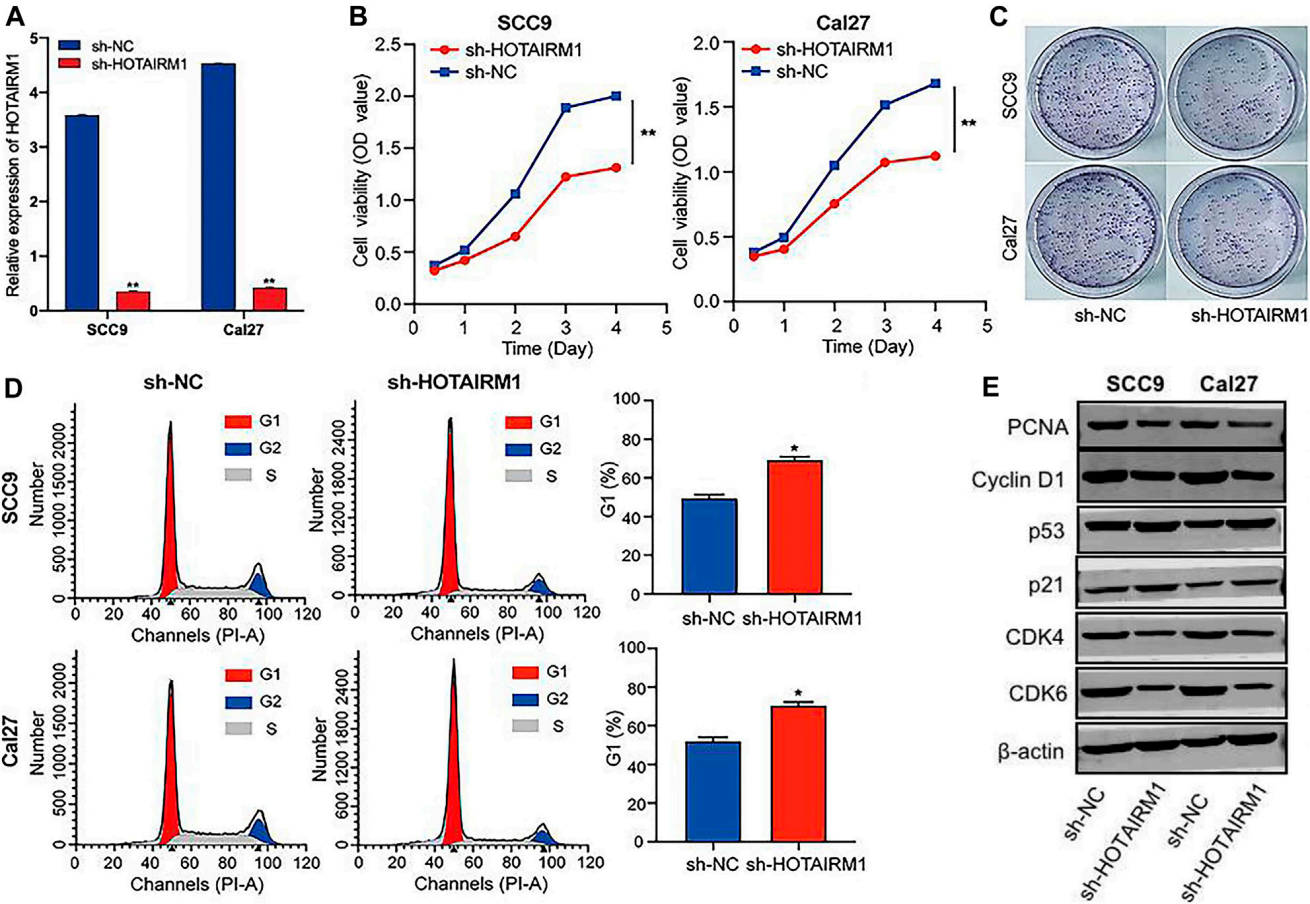
*In vitro* knockdown of HOTAIRM1 restrained cell proliferation and regulated the cell cycle in OSCC cells. **(A)** Knockdown efficiency by sh-HOTAIRM1 in SCC9 and Cal27 cells were validated by qRT-PCR. **(B)** Knockdown of HOTAIRM1 expression in OSCC cells suppressed cell growth measured via the CCK-8 assay. **(C)** Colony formation assays evaluated the influence of HOTAIRM1 knockdown on cell proliferation of OSCC cells. **(D)** Flow cytometry was used to evaluate the impact on cell cycle distribution by knockdown of HOTAIRM1 expression in OSCC cells lines. **(E)** Western blotting showing changes in key biomolecules related to cell proliferation and cell cycle regulation, including PCNA, CyclinD1, p53, p21, CDK4, and CDK6. *p < 0.05, **p < 0.01.

To evaluated the influence on cell proliferation induced by the upregulation of HOTAIRM1 expression in OSCC, we used a sh-HOTAIRM1 strategy to knockdown HOTAIRM1 expression in SCC9 and Cal27 cell lines. The knockdown efficiency of sh-HOTAIRM1 in SCC9 and Cal27 cells were validated by qRT-PCR (Figure 6A), and the results demonstrated that the sh-HOTAIRM1 significantly decreased the expression of HOTAIRM1 in both cell lines compared with the control groups (sh-NC). The follow-up CCK-8 assay indicated that sh-HOTAIRM1 cells presented much slower growth potential than control cells with sh-NC (Figure 6B). Correspondingly, colonies produced by sh-HOTAIRM1 cells showed a weak competitiveness in quantity and size compared with sh-NC cells (Figure 6C). Cell cycle analysis was carried out using flow cytometry to determine whether the inhibition caused by HOTAIRM1 knockdown in OSCC cells was due to a block in the cell cycle. The analysis results indicated a significant G1 phase arrest in SCC9 and Cal27 cells with HOTAIRM1 knockdown (Figure 6D). Furthermore, to provide evidence supporting the role of HOTAIRM1 activity in OSCC at the molecular level, western blotting was performed to evaluate typical regulatory factors and markers associated with cell proliferation and the cell cycle, including the cell proliferation marker PCNA; G1 phase regulatory factor CyclinD1; and key molecules of the cell cycle signaling pathway p53, p21, CDK4, and CDK6. Compared to control groups, the expression of PCNA, Cyclin D1, CDK4, and CDK6 was reduced in sh-HOTAIRM1 OSCC cells, while p53 and p21 expression was markedly increased (Figure 6E). The above results confirmed that the knockdown of HOTAIRM1 induced the inhibition of OSCC cell proliferation, altered the cell-cycle distribution, and arrested cells in G1 phase due to the inhibition of the cell cycle signaling pathway.

### Knockdown of HOTAIRM1 Inhibited Tumor Growth *in vivo*

**FIGURE 7:**
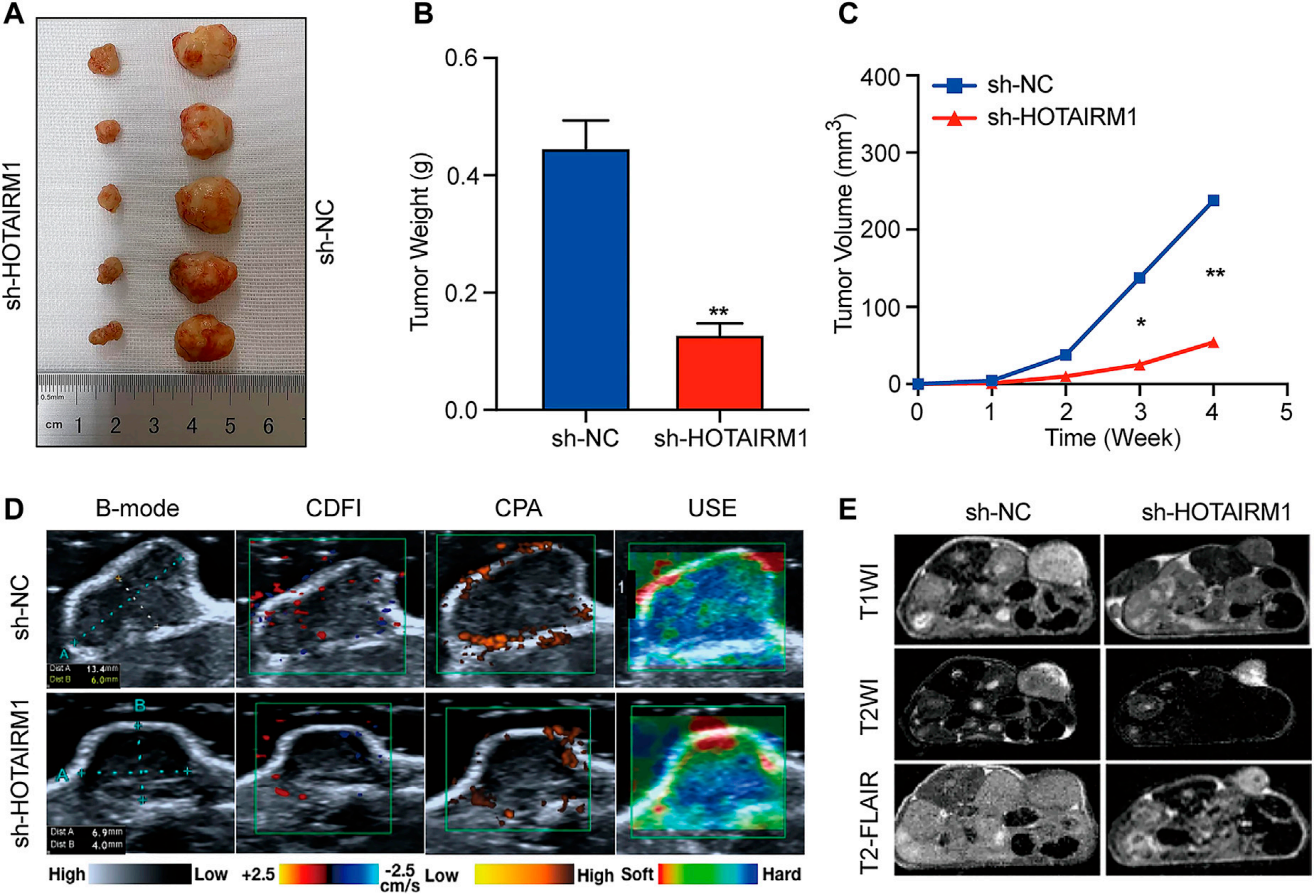
Knockdown of HOTAIRM1 inhibits tumor growth *in vivo*. **(A)** Tumors were excised from nude mice at day 28 after tumor graft inoculation (*n* = 5). **(B–C)** Weight of tumors and Volume were measured and calculated. **(D)** USI evaluation of tumors in mice using B-mode, CDFI, CPA, and USE. **(E)** MRI evaluation of tumors using T1WI, T2WI and T2- FLAIR. *p < 0.05, **p < 0.01.

To investigate the effect of HOTAIRM1 on OSCC *in vivo*, tumor growth of xenografts in nude mice was monitored. HOTAIRM1 knockdown significantly inhibited the growth of xenograft tumors formed by OSCC cells (Figure 7A). As shown in Figures 7B,C, both the tumor weight and volume of the sh-HOTAIRM1 group were obviously smaller than those of the sh-NC group. In addition, the USI and MRI findings provided valuable information on tumor progression and provided information on parameters that are not detected by visual inspection. In sh-HOTAIRM1 groups, both slower growth and slightly weaker internal echogenicity of tumors were observed compared to the control groups, provided by B-mode ultrasonography. Poor angiogenesis and micro-angiogenesis in sh-HOTAIRM1 groups were revealed by Color Doppler Flow Imaging (CDFI) and Color Power Angiography (CPA), respectively. Ultrasonic elastosonography (USE) revealed the slightly weaker hardness of tumors than that in sh-NC groups (Figure 7D). Observed by the MRI, the tumor size of the sh-HOTAIRM1 group was obviously smaller than that of the sh-NC group, which was consistent with the results of USI. Furthermore, in both the sh-NC group and sh-HOTAIRM1 group, the tumors presented low signal intensity in T1WI and high signal intensity in T2WI/T2-FLAIR, that were concordant with the conventional MRI performance of OSCC (Figure 7E). In summary, these findings convincingly demonstrated that HOTAIRM1-knockdown inhibited tumor growth *in vivo*.

## Discussion

As the most common malignant tumor involving the oral and maxillofacial regions, OSCC exerts a severe negative impact on patients due to its poor prognosis and high recurrence rate (Ferlay et al., 2015; Economopoulou et al., 2017; Speight et al., 2017). Currently, the standard measures for treating OSCC involve the surgery to resect the tumor followed by adjunctive therapy such as chemotherapy and radiation. Nonetheless these treatment approaches are not effective in improving the clinical outcome of OSCC patients (Zanoni et al., 2019). Because traditional surgical treatment cannot effectively improve the prognosis of OSCC patients, searching for reliable biomarkers is essential to improve the diagnosis and treatment of OSCC in the early stages of the disease (Li et al., 2020; Jia et al., 2021). Based on previous studies, abnormally expressed lncRNAs have been recognized as moderators of tumor evolution in different cancers, exerting procarcinogenic function or anticancer function by regulating the malignant biological behaviors of tumors directly or indirectly, including processes such as proliferation, migration, invasion, and glycolysis (Schmitt and Chang 2016; Ahadi 2020; Song and Kim 2021). For example, FOXD2-AS1 is upregulated in OSCC and has been reported to inhibit the progression of gallbladder cancer by mediating methylation of MLH1 (Liang et al., 2020). LINC01410 has been reported to be abnormally expressed in cervical cancer and promotes the growth and invasion of cancer cells by regulating the miR-2467/VOPP1 axis (Liu and Wen 2020).

In this study, our aim was to identify the clinically valuable lncRNAs pivotal to the initiation and progression of OSCC and to further elucidate their functions and clinical significance. Our study was the first to determine that HOTAIRM1 was an upregulated lncRNA in OSCC according to the differential expression analysis, which was subsequently confirmed by its high expression in OSCC cell lines, confirming the relevance between HOTAIRM1 expression and OSCC. However, a review of the literatures relative to HOTAIRM1 showed that HOTAIRM1 was previously explored only in thyroid, ovarian cancer, non-small cell lung cancer, leukemia, and clear cell renal cell carcinoma (Zhang et al., 2014; Chao et al., 2020; Hamilton et al., 2020; Chen D. et al., 2021; Li et al., 2021; Liang et al., 2021). In leukemia, HOTAIRM1 was considered to be unfavorable and it could activate RHOA/ROCK1 pathway to enhance glucocorticoid resistance by inhibiting ARHGAP18 (Liang et al., 2021). In ovarian cancer, silencing HOTAIRM1 promoted cell proliferation and inhibited cell apoptosis by regulating the Wnt pathway and its downstream target gene MMP9 (Ye et al., 2021). Thus, it was worthy to further investigate the functional effects of HOTAIRM1 given the lack of studies evaluating HOTAIRM1 in OSCC. We analyzed the correlation between upregulated HOTAIRM1 expression and clinical data of OSCC patients, in which OS and tumor clinical stages were associated with HOTAIRM1 expression, indicating an unfavorable prognosis as well as the prognostic value of HOTAIRM1 in OSCC.

Some scholars proposed that a succession of genomic alterations in neoplastic cells are crucial triggering factors for cancers (Wu and Li 2021), therefore, we further assessed the correlation between HOTAIRM1 expression patterns and tumorigenic characteristics. In our study, the top five mutant genes most related to HOTAIRM1 are TP53, FAT1, MUC16, DNAH5 and HRAS. TP53 gene mutation was found to be most correlated with abnormal expression of HOTAIRM1. Increasing evidence has shown that the mutational status and the functional regulation of TP53 in cancers are related to the disordered expression of lncRNAs directly or indirectly (Aubrey et al., 2018). TP53, is considered the “guardian of the genome” and triggers cell apoptosis by inhibiting multiple pathways, and participates in the identification of DNA damage via DNA repair processes, thereby playing an important role in maintaining genomic stability (Chatterjee and Viswanathan 2021). In other words, disrupting the “guardian of the genome” could force damaged cells into senescence or apoptosis, thereby accelerating the accumulation of somatic mutations. Moreover, FAT1 mutation information has been repeatedly detected in human cancers, especially in squamous cell carcinoma (Mountzios et al., 2014); MUC16 was recognized as the third most common mutant oncogene (Bafna et al., 2010); HRAS was a member of the RAS family of oncogenes, and its mutation has been confirmed to be closely related to the initiation of OSCC (Chai et al., 2020). This was also consistent with our analysis, in which in the high-HOTAIRM1 expression group, the somatic mutation rate was significantly higher than that of the low-HOTAIRM1 expression group. In addition, our study also identified obvious genome amplification and deletion patterns (CNVs) in the high-HOTAIRM1 expression group. Similarly, in anaplastic thyroid cancer (ATC), the amplification of HOTAIRM1 genome copy number increased the expression of HOTAIRM1, and drove the occurrence of ATC by inhibiting the biosynthesis of miR-144 (Zhang et al., 2021). Our study indicated that, at the genetic level, HOTAIRM1 may play a stimulatory role in driving OSCC occurrence.

With the progressive revelation of tumour heterogeneity, cancer stem cells (CSCs) are generally considered to be the components of cancer initiation, which are able to form tumors and influence the progression and malignancy of cancers (Clarke 2019). In Chen’s study of esophageal squamous cell carcinoma, up-regulated LINC-POU3F3 promoted the upregulation of tumor cell stem cell markers (CD133, CD44 and CD90), thereby enhancing the radiotherapy resistance of tumor cells and increasing the degree of malignancies of esophageal cancer (Chen et al., 2021b). mRNAsi is considered to be an indicator to describe the stemness of tumor cells, which could quantify the CSCs to a certain extent (Malta et al., 2018). In our study, the high expression of HOTAIRM1 was associated with higher mRNAsi, indicating that the higher the expression of HOTAIRM1, the stronger the dryness of OSCC cells, suggesting that HOTAIRM1 may promote the malignant potential of OSCC. The TME is described as the environment of tumor cell production and growth, which includes not only the tumor cells themselves but also the surrounding fibroblasts, immune and inflammatory cells, and other cells, as well as the adjacent intercellular signals, microvessels, and relevant infiltrating biological molecules (Balaji et al., 2021). In our study, the HOTAIRM1 expression was inversely proportional to stromal cell and immune cell content, suggesting that HOTAIRM1 may promote the proliferation of OSCC cells and improve tumor purity. Studies accumulating over the past few decades have shown that, tumor-antagonizing areas are often accompanied by chronic inflammation and on the other hand, the infiltration of immune cells in tumor tissues serve to promote tumor evolution (Denaro et al., 2019). To date the identified tumor-promoting immune cells consist of macrophages, mast cells, and neutrophils, as well as T lymphocytes and B lymphocytes, which are confirmed to contribute to induce and help maintain tumor angiogenesis, to promote tumor cell proliferation, and to facilitate metastasis and dissemination *via* seeding of cancer cells at the edge of the tumor (Anderson 2014). In our study, the HOTAIRMI expression was negatively correlated with macrophage M1 (anti-tumor phenotype) and mast cells, and positively correlated with monocytes and eosinophils. Eosinophils infiltrate multiple cancers and have the ability to regulate tumor progression and promote tumor growth either directly by interacting with cancer cells or indirectly by regulating TME (Grisaru-Tal et al., 2020). In the tumor immune microenvironment, monocytes mainly support tumor cells to escape host immune response by infiltrating tumor and differentiating into tumor-related macrophages, thus affecting tumor progression (Chittezhath et al., 2014). In this research, HOTAIRM1 expression levels were correlated with the types of the immune cells, and we speculated that HOTAIRM1 might influence the tumor immune microenvironment and thus promote the progression of OSCC.

To further explore the biological function of HOTAIRM1 in OSCC, WGCNA was used to identify genes most closely related to HOTAIRM1 expression. We determined that nearly all the crucial genes were involved in cell proliferation and the cell cycle, such as G1/S checkpoint, DNA replication, DNA mismatch repair, and DNA damage detection. Subsequently, the function of HOTAIRM1 was verified using GSEA and GSVA. Moreover, *in vitro* cell function experiments showed the inhibition of proliferation and the arrest in G1 phase cell cycle following HOTAIRM1 knockdown in OSCC cells. As a classical cell proliferation marker at the molecular level (Lu et al., 2019), PCNA protein expression was significantly decreased in the sh-HOTAIRM1 knockdown cells, which also suggested that HOTAIRM1 knockdown inhibited the proliferation of OSCC cells. In addition, the detection of molecular markers related to cell cycle pathway showed that p53 and p21 were significantly increased, while the CyclinD1, CDK4 and CDK6 were decreased in OSCC cells with HOTAIRM1 knockdown. The smooth progression of the cell cycle is a guarantee of cell growth and is mainly controlled by p53 via monitoring checkpoints progression at G1/S and G2/M phases, which is closely related to the transcriptional activation of cell cycle-related proteins (Li et al., 2019). As a p53 downstream gene, p21 is a cyclin-dependent kinases (CDKs) inhibitor, which participates in cell cycle processes, plays a key role in tumors activity through the p53 signal pathway, and can bind with a series of cyclin/CDKs complexes to inhibit the activity of corresponding protein kinases, mainly in the G1 phase (Xiong et al., 2021). CyclinD1, a G1/S-specific cyclin-D1, is a marker of G1 phase, and mainly forms complexes with CDK4 or CDK6 to regulate the cell cycle and is indispensable for G1 phase entry to the S phase (Yan et al., 2021). In various cancers, like in lung cancer, gastric cancer, thyroid cancer, and ovarian cancer (Liang et al., 2019; Qiu et al., 2021b; Yan et al., 2021), p53 and p21 jointly constitute the G1 checkpoint of the cell cycle to ensure that the cancer cells at G1 phase smoothly enter S phase. FKBP11 has been described as a regulator of the cell cycle and apoptosis via p53/p21/p27 and p53/Bcl-2/Bax signaling pathways in OSCC, thereby promoting the proliferation of cancer cells (Qiu et al., 2021b). BCAR4 is upregulated in esophageal squamous cell carcinoma acting on the G1 phase of cell cycle to promote cell proliferation by regulating the miR-139–3p/ELAVL1 axis and the p53/p21 signaling pathway (Yan et al., 2021). In human acute promyelocytic leukemia, HOTAIRM1 knockdown inhibits NB4 granulocyte cells differentiation by maintaining cells in the G1 phase and by regulating integrin gene expression levels (Zhang et al., 2014). In brief, our results revealed that in OSCC, the knockdown of HOTAIRM1 upregulated the expression of p53 and p21, maintaining the cells at G1 phase, likely due to the inhibition of the p53/p21-mediated cell cycle signaling pathway, thereby inhibiting cell proliferation. Finally, in xenograft tumor experiments in nude mice, tumor growth was effectively inhibited when HOTAIRM1 was knockdown in OSCC xenografted cells, and resulted in tumors with smaller weight and volume, weaker blood flow signals, and reduced tumor stiffness, as detected using ultrasound imaging and MRI. It is well known that ultrasound imaging and MRI are authoritative tools used to evaluate tumors activity in the clinic (Smiley et al., 2019; Lalfamkima et al., 2021). Altogether, it can be concluded that HOTAIRM1 exhibits tumorigenic properties and facilitates tumor growth in OSCC.

In conclusion, our study was the first to identify HOTAIRM1 as a significantly upregulated lncRNA in OSCC, which is closely related to poor prognosis of patients and may represent a new potential biomarker for OSCC. Moreover, HOTAIRM1 was shown to be carcinogenic in OSCC, by promoting cell proliferation and by accelerating cell cycle progression, via regulatory factors in the p53/p21 pathway induced by abnormal HOTAIRM1 expression. Overall, our study revealed that HOTAIRM1 acts as a novel possible prognostic biomarker and therapeutic target for OSCC.

## Data Availability

The original contributions presented in the study are included in the article/Supplementary Material, further inquiries can be directed to the corresponding authors.

## Abbreviations

OSCC, oral squamous cell carcinoma; HNSC, head and neck squamous carcinoma; lncRNAs, long non-coding RNAs; CNV, copy number variation; TMB, tumor mutation burden; TME, tumor microenvironment; GO, Gene Ontology; KEGG, Kyoto Encyclopedia of Genes and Genomes; WGCNA, Weighted gene co-expression network analysis; GSEA, Gene set enrichment analysis; GSVA, Gene set variation analysis; USI, Ultrasonic imaging; MRI, magnetic resonance imaging.
