# Supplementary figures and images for: Identification and Validation of HOTAIRM1 as a Novel Biomarker for Oral Squamous Cell Carcinoma
Source: Front Bioeng Biotechnol. 2022 Jan 11;9:798584. doi: 10.3389/fbioe.2021.798584 (PMC8787327; doi:10.3389/fbioe.2021.798584)

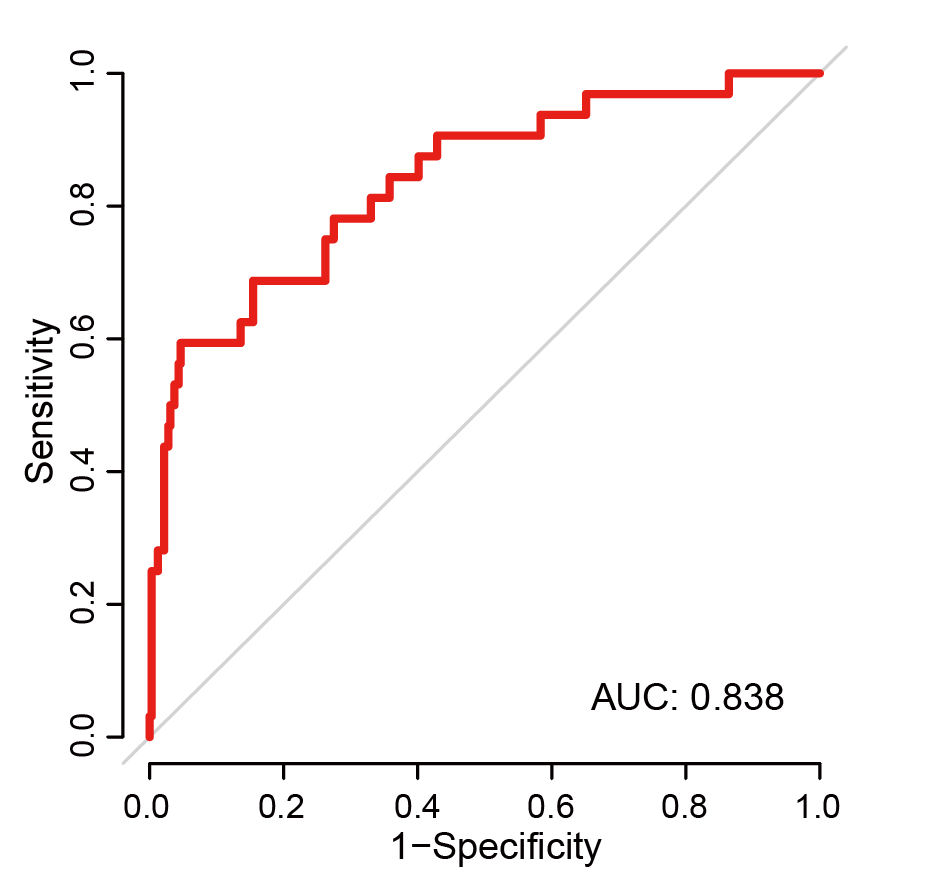

Supplement: Supplementary file 3 [file Image1.JPEG]
